# Textbook of Global Health, Fourth Edition

**Published:** 2017-04-20

**Authors:** Lori Hanson

**Affiliations:** 1 Department of Community Health and Epidemiology, University of Saskatchewan, Saskatchewan, Canada

As with the first three editions of the *Textbook of Global Health*^1^ *(*formerly *Textbook of International Health)*, the highly integrated, interdisciplinary, comprehensive, and unique presentation of the subject matter makes this book at once a serious and essential contribution to teaching and learning Global Health. The *Textbook* provides a thoroughly researched, logically laid out plan for either a novice or seasoned teacher, as it is conveniently divided into the number of sessions typically required in a semester long course. The busy upper year medical undergrad or graduate student is contemplated in its design, as each of the fourteen chapters includes an overall introduction, key questions, concepts, examples and an appropriate number of legible and clearly labeled figures and diagrams. The stories and examples - often set apart in textboxes - are reader friendly, audience appropriate, and engaging. They embellish the text, but are not essential, allowing for readings of just core content, or for deeper insights into history and practice.

The chapters are laid out logically, beginning with the historical origins of international health and its transition to (and continuity with) global health. The book continues with the building blocks of required knowledge in the field including: global health actors and activities, data and epidemiological profiles, and an introduction to the political economy of health. The middle chapters of the book frame, and then discuss, central concepts and issues of importance to the field: health equity and the social determinants of health; crises and humanitarianism; globalization, trade and health; health and the environment; health care systems; and health economics and financing. The penultimate chapter describes specific interventions including case studies of healthy societies. The last chapter suggests promising forms of alternative global health practice as captured in its title: “Towards a social justice approach to global health.” If a student enters a global health course using this textbook with little interest in the subject, they will leave passionate and emboldened to embark upon the challenges in a career and/or commitment to the field. Still, the book is not for the faint of heart and requires serious academic pursuit. With 646 pages of text, this is not a book to be whipped off in a weekend of cramming at the end of the semester. The *Textbook’s* authors make visible from whence such an integrated volume might arise. With a passion and talent for digging in historical records, and having published extensively in journals on four continents, Anne-Emanuelle Birn is a Professor of Critical Development Studies and Social and Behavioural Sciences at the University of Toronto, where she served as a Canada Research Chair in International Health for ten years. Yogan Pillay, with 20 years of planning and implementation practice in health systems reforms, is the current Deputy Director General for HIV, Tuberculosis and Maternal, Newborn, and Child Health programs in the National Department of Health in South Africa. Founder of the health and social justice organization Doctors for Global Health, Timothy Holtz is an expert in infectious diseases epidemiology and disease control and former consultant to the CDC and WHO, who currently sits as Adjunct Professor of Global Health at the Rollins School of Public Health at Emory University.

Having taught Global Health for the past 16 years, and having concluded that much of its practice is as problematic as its predecessor, International Health, it is refreshing and invigorating to see a text engage with the contradictions of its subject with such rigor and honesty. It is anything but simple to connect the dots between the individual cases of merciless poverty and disease that are rife in the world with macro-economic policies and political systems that create and perpetuate those conditions. So very many Global Health textbooks provide epidemiological, bio-medical and/or behavioural paradigms for its study – but only this one takes those perspectives to the next level, offering its reader a framework of understanding to make those connections through the systems lens of political economy. Yet, throughout the text – to lesser and greater degrees depending on the chapter - the authors skillfully integrate and critique the shortcomings of bio-behavioural approaches to health problems without dismissing them. With similar sophistication, they include the perspectives of different disciplines as ways of disentangling the web of causation of disease and inequities. Economics, sociology, medicine, nursing, public health, social work, anthropology, political science, history and ecology are all invoked.

No work of such ambitious breadth can ever be complete and nor would this text withstand much expansion without causing student revolt, but I would propose two additions: one, to improve its graphic appeal, being the addition of more visual data (photos, drawings, etc.); and the second, the addition of a separate more robust section or chapter on issues related to colonialism and the health of indigenous peoples – perhaps with a subsection on settler colonialism and indigenous peoples in High Income Countries.

Students embarking on a course using the *Textbook* may have entered the field of Global Health with certain ideas based on popular characterizations of the field. Cognizant of the good will, humanitarian impulse, and sometimes missionary zeal with which students sometimes simplistically envision the practice of global health, the authors are sympathetic in their critique of popular, though harmful and inequitable practices. Carefully leading their readers to question their place in the world, they instead add a refined fuel to the fires in the bellies of the protégés, eventually enticing their critical and ethical involvements and de-centering the Western medical expert role, while growing understanding of the necessarily more complex, equitable engagements unfolds. My favourite chapter in the *Textbook* is the last, for in its aspirations for social justice we encounter a critically refreshing perspective with alternative routes toward equitable global health.
